# Nutritional Surveillance for the Best Start in Life, Promoting Health for Neonates, Infants and Children

**DOI:** 10.3390/nu12113386

**Published:** 2020-11-04

**Authors:** Valeria Calcaterra, Hellas Cena, Elvira Verduci, Alessandra Bosetti, Gloria Pelizzo, Gian Vincenzo Zuccotti

**Affiliations:** 1 Pediatric and Adolescent Unit, Department of Internal Medicine, University of Pavia, 27100 Pavia, Italy; 2 Department of Pediatrics, “V. Buzzi” Children’s Hospital, 20157 Milano, Italy or elvira.verduci@unimi.it (E.V.); alessandra.bosetti@asst-fbf-sacco.it (A.B.); or gianvincenzo.zuccotti@unimi.it (G.V.Z.); 3 Laboratory of Dietetics and Clinical Nutrition, Department of Public Health, Experimental and Forensic Medicine, University of Pavia, 27100 Pavia, Italy; hellas.cena@unipv.it or; 4 Clinical Nutrition and Dietetics Service, Unit of Internal Medicine and Endocrinology, ICS Maugeri IRCCS, 27100 Pavia, Italy; 5 Department of Health Sciences, University of Milano, 20142 Milano, Italy; 6 Department of Biomedical and Clinical Science “L. Sacco”, University of Milano, 20157 Milano, Italy; gloria.pelizzo@unimi.it or; 7 Pediatric Surgery Unit, “V. Buzzi” Children’s Hospital, 20157 Milano, Italy

**Keywords:** maternal, nutrition, newborn, fetus, promotion, children, health

## Abstract

This Special Issue aims to examine the crucial role of nutritional status starting from pregnancy in modulating fetal, neonatal and infant growth and metabolic pathways, with potential long-term impacts on adult health. Poor maternal nutritional conditions in the earliest stages of life during fetal development and early life may induce both short-term and longer lasting effects; in particular, an increased risk of noncommunicable diseases (NCDs) and other chronic diseases such as obesity, which itself is a major risk factor for NCDs, is observed over the lifespan. Poor maternal nutrition affects the fetal developmental schedule, leading to irreversible changes and slowdown in growth. The fetus limits its size to conserve the little energy available for cardiac functions and neuronal development. The organism will retain memory of the early insult, and the adaptive response will result in pathology later on. Epigenetics may contribute to disease manifestation affecting developmental programming. After birth, even though there is a limited evidence base suggesting a relationship between breastfeeding, timing and type of foods used in weaning with disease later in life, nutritional surveillance is also mandatory in infants in the first year of life. We will explore the latest findings on nutrition in early life and term and preterm babies, as well as the role of malnutrition in the short- and long-term impact over the lifespan. Focusing on nutritional interventions represents part of an integrated life-cycle approach to prevent communicable and non-communicable diseases.

This Special Issue aims to examine the crucial role of nutritional status starting from pregnancy in modulating fetal, neonatal and infant growth and metabolic pathways, with potential long-term impacts on adult health.

Poor maternal nutritional conditions at the earliest stages of life during fetal development and early life can induce both short-term and longer lasting effects; in particular, an increased risk of noncommunicable diseases (NCDs) and other chronic diseases such as obesity, which itself is a major risk factor for NCDs, is observed over the lifespan [1,2,3,4,5].

According to the hypothesis of the “developmental origins of health and disease” (DOHaD), proposed by Barker in the late 1980s, there is a relationship between unfavorable fetal conditions and the development of diseases in adulthood and older age [6,7,8,9,10]. Humans demonstrate plasticity during their fetal development, and adverse conditions in the early embryonic organogenetic phase during development may permanently change the structure of organs and systems according to the so-called phenomenon of “fetal programming”.

Fetal development is characterized by rapid growth whose main feature is cell division, as well as cell, tissue and organ differentiation. These so-called “critical” periods are short periods, which occur at different times for different organs or systems. Despite the fact that recent evidence has highlighted the importance of the preconception period on fetal programming [11,12], much of the biological development is completed in the first 1000 days from conception.

One of the most impairing adverse conditions for fetal development is represented by malnutrition. Both under-nutrition and over-nutrition deserve special attention since they are both characterized by nutrient imbalance, both macro- and micro-nutrients).

Poor maternal nutrition affects the fetal developmental schedule, leading to irreversible changes and slowdown in growth (“thrifty phenotype”). One of the main consequences of nutrient deficiencies is cell division slowdown in those tissues that are at a critical point. The fetus limits its size to conserve the little energy available for cardiac functions and neuronal development. The product of conception is programmed to respond to a “poor” and hostile environment, while during childhood and adulthood, individuals lose their “plasticity”, becoming unable to adapt to a more “rich” environment, and they are therefore exposed to an increased disease risk [6,7,8,9,10], including NCDs (type 2 diabetes, cardiac disorders, cancers, and chronic respiratory disease).

The mechanisms by which environmental insults disrupt fetal development are not fully understood; nevertheless, there is no doubt that an adverse environment in utero caused by maternal malnutrition not only impacts fetal development, subsequently affecting offspring outcome, preterm births, inadequate intrauterine growth rates, congenital defects, and other complications, but also leads to epigenetic modifications, which have a key role in “fetal programming” [11]. By means of epigenetics, we began to understand how nutrients in the prenatal environment are transmitted to the fetus, inducing a new "reprogrammed" phenotype. Since nutrients are necessary for the methylation process, initially, factors such as nutrition, but also other lifestyle and environmental factors, including physical activity, sleep patterns, exposure to smoke, medication, and endocrine disruptors chemicals, alter epigenetic patterns [12,13]. These alterations, if maintained for a long time, may affect gene expression and cause changes in phenotypic traits. Prevention and understanding of epigenetic damage processes are fundamental since nutrition has trans-generational epigenetic effects.

After birth, even though there is a limited evidence base suggesting the relationship between breastfeeding, timing and type of foods used in weaning with disease later in life, nutritional surveillance is also mandatory in infants in the first year of life [14]. A number of studies suggest the beneficial role of breastfeeding on immune and neurocognitive development and its protective effects against obesity, diabetes and hypertension [15,16]. The impact of complementary feeding timing and modality on later onset of NCDs should be also considered in life-long health status outcome [16].

Special attention to nutrition should also be paid for preterm infants. Preterm birth contributes to neonatal morbidity and physical and neurodevelopmental disabilities. Additional long-term health consequences of preterm birth, such as an increased risk of hypertension and insulin resistance in adult life, are influenced by early nutrition and infant and childhood growth rates [17].

In conclusion, nutritional surveillance in pregnancy, early life and infancy is crucial in promoting the best start in life, inducing short- and long-term positive effects on children’s health, and reducing the social healthcare costs associated with malnutrition-related diseases. In this issue, we will explore the latest findings on nutrition in early life and term and preterm babies, as well as the role of malnutrition in the short- and long-term impact over the lifespan. Focusing on nutritional interventions represents part of an integrated life-cycle approach to prevent communicable and non-communicable diseases.
